# Absence of changes in the milk microbiota during *Escherichia coli* endotoxin induced experimental bovine mastitis

**DOI:** 10.1186/s13567-023-01179-5

**Published:** 2023-06-08

**Authors:** Josef Dahlberg, Carl-Fredrik Johnzon, Li Sun, Gunnar Pejler, Karin Östensson, Johan Dicksved

**Affiliations:** 1 Department of Animal Nutrition and Management, Swedish University of Agricultural Sciences, Uppsala, Sweden; 2 Department of Anatomy, Physiology and Biochemistry, Swedish University of Agricultural Sciences, Uppsala, Sweden; 3 Department of Molecular Science, Swedish University of Agricultural Sciences, Uppsala, Sweden; 4 Department of Clinical Sciences, Swedish University of Agricultural Sciences, Uppsala, Sweden; 5 Department of Medical Biochemistry and Microbiology, Uppsala University, Uppsala, Sweden

**Keywords:** Milk microbiota, contamination, low biomass, 16S sequencing

## Abstract

Changes in the milk microbiota during the course of mastitis are due to the nature of a sporadic occurring disease difficult to study. In this study we experimentally induced mastitis by infusion of *Escherichia coli* endotoxins in one udder quarter each of nine healthy lactating dairy cows and assessed the bacteriological dynamics and the milk microbiota at four time points before and eight time points after infusion. As control, saline was infused in one udder quarter each of additionally nine healthy cows that followed the same sampling protocol. The milk microbiota was assessed by sequencing of the 16 S rRNA gene and a range of positive and negative controls were included for methodological evaluation. Two different data filtration models were used to identify and cure data from contaminating taxa. Endotoxin infused quarters responded with transient clinical signs of inflammation and increased SCC while no response was observed in the control cows. In the milk microbiota data no response to inflammation was identified. The data analysis of the milk microbiota was largely hampered by laboratory and reagent contamination. Application of the filtration models caused a marked reduction in data but did not reveal any associations with the inflammatory reaction. Our results indicate that the microbiota in milk from healthy cows is unaffected by inflammation.

**Supplementary Information:**

The online version contains supplementary material available at 10.1186/s13567-023-01179-5.

## Introduction

Experimentally induced mastitis, obtained as a response, either to infection with live bacteria or to bacterial endotoxins has been used as a model to study various pathological, immunological and microbial reactions during the course of mastitis [1–8]. It has previously been shown that intra-mammary infusion of *Escherichia coli* bacteria or its endotoxin (lipopolysaccharide, LPS) leads to a pronounced inflammatory reaction with an increase in concentration of neutrophils [6], and various soluble bacteriostatic and immunological factors [6, 7, 9–12]. The neutrophils’ phagocytic and detoxification capacity plays the outmost important role for bacteria and toxin clearance in mastitis. Antibiotic treatment in *E. coli* mastitis is doubtful. It has been shown to neither improve the cure rate in spontaneous acute mastitis [13] nor affect the pathogen clearance during an experimental infection [5].

The idea of an always present bacterial community in bovine milk is new, controversial and contradictory to the dogma that milk from healthy individuals is produced sterile and that presence of microbes in milk from healthy mammary glands, when cultured according to mastitis diagnostic routines, is a result of contamination during sampling. Studies using next generation sequencing techniques to investigate bovine milk microbiota have concluded that there is a microbiota present in milk from healthy mammary-gland quarters and that the diversity of the microbiota is higher in healthy quarters compared to clinically or sub-clinically infected quarters [14]. Further, it has been shown that the bacteria found in milk from an infected quarter by culturing is not always the most commonly occurring bacteria by DNA sequencing methods [15]. It has also been shown that bacterial diversity varies with somatic cell count (SCC), which is the most commonly used marker of inflammation for bovine mammary health [16]. The microbiota in milk has also been studied and associated to history of intra-mammary infection [17], cow genotype [18] and farm environment [19]. However, the idea of a microbiota in bovine milk has been challenged due to its incompatibility with current knowledge about mammary gland immunology and mastitis control plans [20]. The milk microbiota in naturally occurring cases of *E. coli* mastitis has been described and associated with linear score (an SCC-based udder health metric), milk production and the effect of antibiotic treatment. In addition, studies of experimentally induced mastitis with live *E. coli*, as well as spontaneous cases of *E. coli* mastitis have indicated that the microbial diversity of the milk microbiota, measured by Shannon diversity index, was decreased at identification of disease or during peak inflammation [5, 21, 22].

Sequencing of amplicons generated from the 16 S rRNA gene has become the most commonly used technique to describe bacterial communities in various environments during the past decade. A technical challenge with 16 S amplicon-sequencing is that the technique is prone to introduction of biases (see Pollock for review [23]) and results can vary with type of primers used [24]. Another problem is that samples with low bacterial biomass have a lower reproducibility [25] and are easily contaminated. Laboratory and reagent contamination has been shown to largely affect the results in microbiota studies where the bacterial biomass is low [26–29]. As a consequence, methods to identify and cure data from contamination has been developed [30–33].

In this study we describe the bovine milk microbiota during the course of experimentally induced endotoxin mastitis. Our hypothesis was that (a) there is a microbiota in milk from healthy mammary glands that is affected by inflammation, and (b) that filtration of potential contaminating taxa might be needed in order to establish differences related to inflammation in the milk microbiota.

## Materials and methods

### Animals and experimental setup

Animal management and the sampling procedure have previously been described in detail by Johnzon et al. [4]. In brief, the experiment was conducted in two rounds with identical setup. In the first round, eight primiparous cows were included and in the second round ten primiparous cows. In each round, 50% of the animals were infused with 100 µg purified LPS (*Escherichia coli* serotype O111:B4, Sigma-Aldrich, Saint Louis, USA) dissolved in 10 mL physiological saline solution (0.9% NaCl for parental use, Braun, Melsungen, Germany) in one selected quarter, through the teat canal. Control animals were infused in one selected quarter with 10 mL physiological saline solution, (0.9% NaCl for parental use, Braun, Melsungen, Germany), cows were randomly allocated to receive either LPS or NaCl. Healthy primiparous cows housed in a loose housing system at The Swedish Livestock Research Centre were recruited to the study. For easier sampling and better control of animal welfare, cows were moved to a tie-stall unit within the barn 24 h before infusion and returned to the loose housing system 24–48 h after infusion. Sampling of milk was performed on four occasions before infusion and on eight occasions after infusion, corresponding to 144, 96, 48 and immediately (“0 h”) before infusion and 1, 2, 4, 24, 72, 120, 168 and 336 h after infusion. Milk samples from 144, 96 and 48 h before infusion were collected from all four quarters and the quarter with lowest cell count from these sampling points was select to be infused. Before milk sampling, the teat was cleaned with a moist udder cloth, the teat end sterilized with 70% ethanol and 3 squirts of milk was discarded. 25 mL of milk were collected by hand milking into sterile 15 mL tubes and placed in an ice filled cooler directly after sampling. The milk samples were transported to the laboratory, brought to room temperature, gently homogenized and aliquoted into sterile 2 mL tubes and frozen in -80 °C within 10 h, until further use. For SCC analysis, one aliquot of 10 mL milk was mixed with 30 µL 10% bronopol solution (Sigma-Aldrich, Saint Louis, USA) and stored in 4 °C until analysis. The SCC was assessed by an automatic milk somatic cell counter (Fossomatic 5000 from FOSS, Hillerød, Denmark – in the first round and a FTIR 300HP from Perten Instruments, Stockholm, Sweden – in the second round). The Uppsala Ethics Committee approved the study protocol with ref. no. C102215/15 and C22/15.

After sampling, the local inflammatory reactions were assessed by clinical scoring and body temperature was measured. The udder was examined by visual observation and palpation and clinical signs (heat, pain, redness and swelling) were categorized as absent, mild, moderate or severe, receiving a score of 0–3. To determine changes in milk appearance, a small volume was deposited into a black bottomed vessel, and changes were visually evaluated. All scoring and milk sampling was performed by the same member of our research team, the sampler was wearing nitrile gloves that were changed between cows and when dirty.

### Bacterial culturing

In the second round, growth of udder pathogens in milk was assessed by culturing. From each samples 10 µL and 100 µL of milk were cultured on agar plates with 5% bovine blood and 0.05% esculin (National Veterinary Institute, Uppsala, Sweden) in accordance with the National Mastitis Council recommendations for mastitis diagnostics (10 µL) [34] and for increased sensitivity (100 µL). Agar plates were incubated aerobically at 37 °C, growth was evaluated after 24 and 48 h. Agar plates inoculated with 10 µL of milk and with growth of > 2 colony forming units (cfu) were sent for evaluation and MALDI-TOF typing, when appropriate, at the ISO 17,025 accredited Mastitis Laboratory at the National Veterinary Institute (Uppsala, Sweden). Milk on agar plates with ≤ 2 cfu were judged as emanating from noninfected quarters, based on the rules for bacteriological mastitis diagnostics. For agar plates inoculated with 100 µL of milk, the total number of cfu were counted. Milk aliquots were processed and bacterial culturing was performed on an ethanol-cleaned bench top. Only sterile equipment was used in contact with milk.

### DNA preparation and sequencing

All milk samples from LPS-infused quarters and control quarters collected in the experiment (round 1 and 2) were included in sequencing. Milk aliquots were thawed, warmed to room temperature and vortexed before 1 mL of milk was withdrawn for DNA extraction. The milk was centrifuged at 13 000 *g* for 5 min, the skim milk fraction was removed and DNA was extracted from the cell pellet and fat layer using the DNeasy PowerFood Microbial kit, (Qiagen, Hilden, Germany, LOT no. 157,017,245). DNA extraction was performed according to the manufacturer’s instructions, except that a Precellys24 (Bertin Technologies, Montigny-le-Bretonneux, France, 2 × 45 s at 6500 rpm) was used for cell lysis. In parallel, an empty vial was used as a no-template DNA extraction control (NTC) into which the first reagent was added and further processed as the milk samples. In total, 12 NTC were included in DNA extraction. Five commonly occurring mastitis pathogens (*Staphylococcus aureus*, *Streptococcus dysgalactiae*, *Trueperella pyogenes*, *Escherichia coli*, *Klebsiella pneumoniae*) were chosen to create a bacterial mock community used for method evaluation as previously described [26]. The mock community was created with equal numbers of cells and prepared in three different dilutions (10^7^, 10^5^ and 10^3^ cells per mL of each bacterial species) then submitted for DNA extraction in duplicates. DNA from mock communities was extracted using the same protocol as the milk samples. In addition, a DNA-based mock community (ZymoBIOMICS Microbial Community DNA Standard, Zymo Reseach, Irvine, USA) containing DNA from eight different bacterial species was included in the PCR reactions as a second positive control.

Extracted DNA from milk samples, NTC’s, the bacterial mock communities and the DNA-based mock community were subjected to PCR amplification and used to prepare the sequencing library. The Illumina MiSeq sequencing library was prepared by amplifying the V3–V4 region of the 16 S rRNA gene, using the 341 F-805R primers described by Hugerth et al. [35]. The primers contained a linker sequence compatible with barcoding primers that were used to attach sample specific barcodes and Illumina adaptors in a second PCR. Each PCR reaction contained 12.5 µL of DreamTaq PCR master mix (2X) (Thermo Fisher, Waltham, USA), 1.25 µL of each primer in a 10 µM solution, 7 µL DNA-free water and 3 µL of DNA template. Thermocycling was performed on a MyCycler (Bio-Rad Laboratories Inc., Hercules, USA) and thermocycling conditions were: initial denaturation at 94 °C for 3 min, 35 cycles of denaturation at 94 °C for 40 s, annealing at 58 °C for 40 s and elongation at 72 °C for 60 s; and a final elongation at 72 °C for 7 min. All samples were subjected to PCR amplification and re-run once if no band was visible when analysed on 1% agarose gel. All DNA extraction and first PCR preparations were performed in a laminar air-flow hood cleaned with 10% bleach, 70% ethanol and UV-irradiated for 30 min before execution.

Twenty microliters of PCR products were purified with Ampure Beads (Beckman Coulter, Brea, USA) using 0.9 volumes of beads per volume of PCR product and eluted in 40 µL of DNA-free water. The second PCR attaching Illumina adapters and barcodes was performed using the same thermocycling conditions for 10 cycles but with 1 µL of each primer (10 nM solution) and 5 µL of purified PCR products as DNA template. One barcode combination per sample was used. PCR products were again purified with Ampure Beads 0.7 volumes of beads per volume of PCR product and eluted in Elution Buffer. DNA was quantified with Qubit 3.0 Fluorometer (Life Technologies, Carlsbad, USA), the samples were thereafter pooled into equimolar amounts. The DNA pool was concentrated by mixing a large volume of PCR product with Ampure Beads (ratio 1:0.9) and eluted in a smaller volume. The DNA pool was then cleaned through gel extraction (GeneJET, Gel Extraction Kit, Thermo Fisher, Waltham, USA) to ensure that DNA strands of right length was sequenced. The DNA was sequenced on an Illumina MiSeq sequencer with v3 sequencing chemistry, 2 × 300 bp with 10% PhiX (Illumina Inc., San Diego, USA) at the Science for Life Laboratory (Solna, Sweden). Raw sequences have been submitted to the National Center for Biotechnology Information under Bioproject accession number PRJNA580390. Twelve negative PCR reaction controls with no visual bands on agarose gel from the first PCR were included and barcoded in the second PCR. All of the 12 PCR controls (negPCR) contained DNA, when measured with Qubit Fluorometer, and were included in the sequencing.

### Illumina sequence data analysis

Data from the sequencing run was processed as follows: Raw sequencing reads were demultiplexed and analyzed using Quantitative Insight Into Microbial Ecology 2 software (QIIME2, version 2019.4) [36]. Briefly, Cutadapt was used to trim the primer [37]. DADA2 was used as a quality filtering method to denoise, de-replicate paired-end sequences, remove chimera sequences and construct a table of amplicon sequence variants (ASVs) [38]. Taxonomy classification was performed using the QIIME2 naive Bayes classifier trained on 99% Operational Taxonomic Units (OTUs) from the SILVA rRNA database (release 132) [39] after trimming to the primer region. The ASV table was randomly subsampled to contain 10 000 reads per sample.

A data set with taxonomy assigned to genus level, or lowest taxonomy level possible, was used for further analyses. For easier reading “bacterial taxa” are used throughout in the continuation of this text.

#### Statistical analysis

Descriptive analysis on sequencing results, statistical calculations and multivariate analyses were performed using Microsoft Excel 2016, PAST [40] and R [41]. Statistical significance was set at the level *P* < 0.05. After a primary data analysis, two different models were used to identify contamination and filter data. In data filtration model 1 we used the R-package “decontam” [30] to identify contamination, using default settings for method “either”. In addition, unexpected bacterial taxa that appeared in the sequenced mock community were removed from the data set, i.e. bacterial taxa that appeared in the data set but were not included in the mock community. In data filtration model 2 identification of contaminants was based on relative abundance of bacterial taxa in a blank controls. Any bacterial taxa that was present with more than 1% abundance in a NTC or negPCR were classified as contamination and removed from the data set [26].

Principal coordinate analysis (PCoA) and analysis of similarity (ANOSIM) based on Bray Curtis distances and Bonferroni corrected p-values were used to identify clustering patterns among the milk samples before and after data filtration. Separate clustering of samples that responded to the intervention were further explored by comparing the relative abundance of bacterial taxa in groups of samples. Univariate tests were used to test for differences between groups of samples and were evaluated using t-test when comparing two groups, and Mann Whitney pairwise post-hoc test with Bonferroni corrected *p*-values when comparing three or more groups.

## Results

### Animals and clinical signs

**Figure 1:**
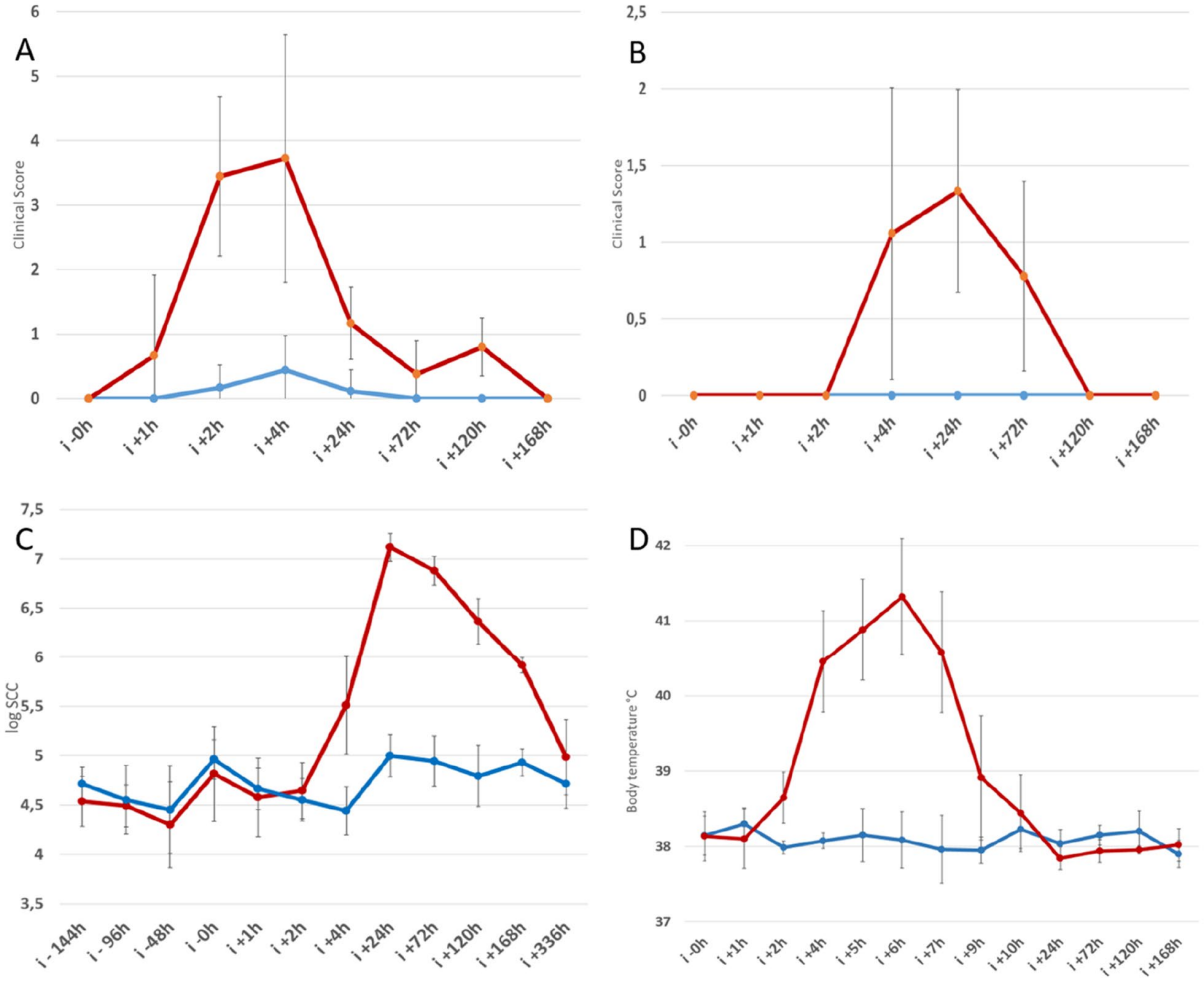
**Summary of clinical and immunological response.** Panel 1 **A** shows local inflammation score i.e. heat, redness, swelling and pain, 1 **B** score of changes in milk appearance, 1 C logSCC per sampling point and 1D body temperature after infusion. For all panels; values are presented as mean ± SD, LPS infused cows are in red (*n* = 9) and control cows are in blue (*n* = 9).

Animals used and the systemic and local signs of illness as a response to the LPS infusion have been described by Johnzon et al. [4]. In short, LPS-infused cows were on average 31.7 months old (range 29–35) and had on average been milking for 178 days (range 133–247), control cows were on average 30.9 months old (range 27–35) and had on average been milking for 143 days (range 26–194). The LPS-infused quarters had a mean somatic cell count of 25 100 cells/mL (range 5000-77 000 cell/mL) during the three sampling points before infusion and the control quarters had a mean somatic cell count of 39 800 cells/mL (range 5000 − 127 000 cells/mL) at the same sampling points. The LPS-infused cows responded with local clinical signs of mastitis (heat, pain, swelling and redness) that were observed from two hours after infusion and lasted 24–72 h. Changes in milk colour and appearance were visible from 4 h post-infusion until 72 h post-infusion. The body temperature of the LPS infused cows started to increase after two hours, peaking with temperatures over 41 °C at 5 h and then returning to normal levels within 24 h post-infusion. The SCC was increased after two hours, peaked with levels > 13 × 10^6^ at 24 h and remained elevated for 168 h. A summary of clinical signs is presented in Figure 1. The control quarters, infused with sterile saline, did not show any local or systemic signs of mastitis.

### Bacterial culturing

One hundred twenty milk samples from 10 udder quarters in 10 cows were subjected to bacterial culturing. The proportion of samples with bacterial growth varied with the amount of inoculated milk. In total, 25% (*n* = 30) and 63% (*n* = 75) of samples were culture positive (i.e. had at least one cfu) after 48 h when using 10 µL or 100 µL of milk, respectively. Seven milk samples had growth of more than 2 cfu after culturing 10 µL of milk and were subjected to a routine mastitis diagnostic evaluation at the Mastitis Laboratory at the National Veterinary Institute. All seven samples were judged as having growth of mixed bacterial flora, i.e. growth of three or more bacterial species on the agar plate, based on colony morphology.

During the time period from four to 168 h post-infusion, when mean SCC was > 200 000 cells/mL in LPS-infused quarter, the number of culture positive samples from these LPS-infused quarters was numerically lower than from control quarters (6 vs. 8 culture positive samples when using 10 µL of milk and 12 vs. 20 culture positive for 100 µL of milk).

### Sequencing results

**Figure 2:**
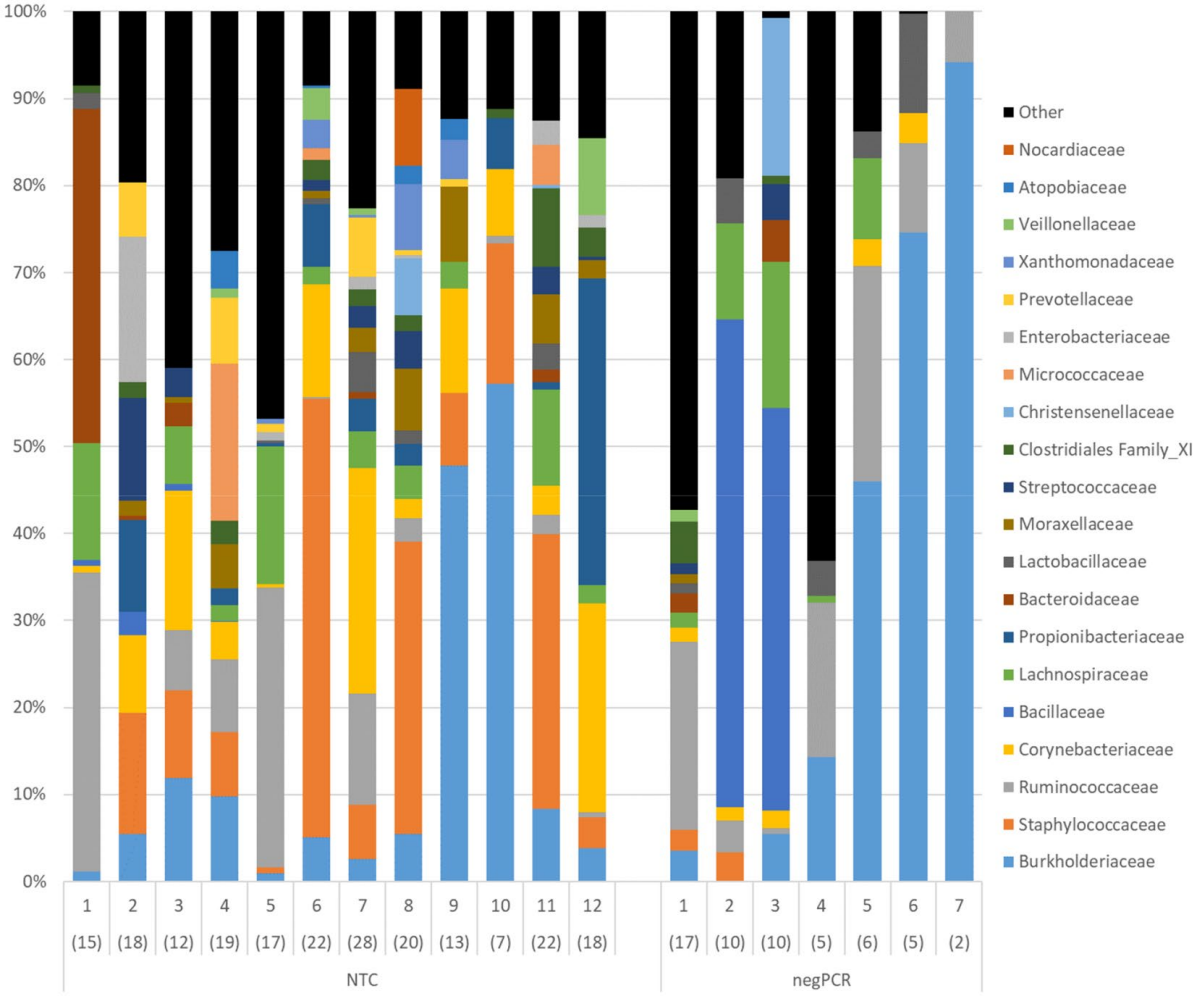
**Distribution of the 20 most abundant taxonomic families in no template DNA extraction controls (NTC) and negative PCR controls (negPCR) after sequencing.** Number within parenthesis equals’ number of identified families per sample.

In total, 209 milk samples were submitted for sequencing together with 12 NTC, 12 negPCR and 8 positive controls. One negPCR and one milk sample failed to generate any reads. The milk samples generated on average 19 081 ± 6838 quality controlled reads per sample, the NTC’s generated on average 32 390 ± 10 193 reads per sample, the negPCR generated on average 13,154 ± 6335 reads per sample and the mock communities generated on average 20 573 ± 5277 reads per sample, the total number of reads were 4 666 764. The number of reads per sample in the NTC’s were significantly higher than the other groups (*p* < 0.05, Mann Whitney pairwise). With the rarefaction level set at 10 000 reads per sample, 217 samples were used for further analyses (190 milk samples, 8 mock community samples, 12 NTC and 7 negPCR). The remaining samples had fewer than 10 000 reads and were thus excluded from further analyses. In total, 389 different bacteria taxa were identified in the data set. The negative controls (NTC and negPCR) contained 129 different taxa and 99 of those were present with more than 1% abundance in a negative control sample. The distribution of the 20 most abundant families in the sequenced negative controls is presented in Figure 2 and a complete list of bacterial taxa identified in negative controls is provided in Additional file 3.

### Sequenced positive controls

**Figure 3:**
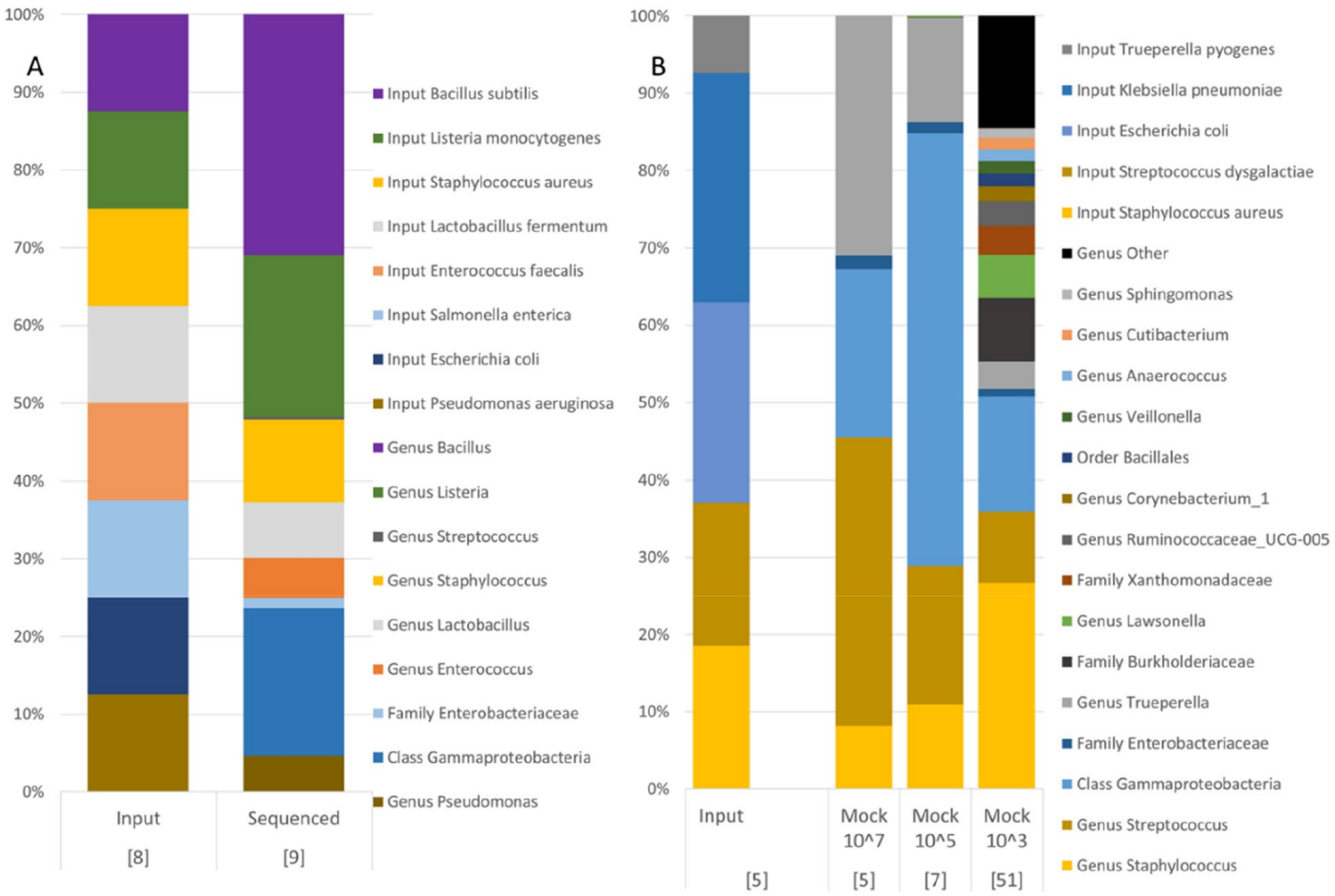
**Theoretical abundance in mock communities and relative abundance of the 15 most abundant bacterial taxa in mock communities after sequencing.** Panel **A** shows input and sequenced output from the DNA based mock community whereas panel **B** shows input and sequenced output from the bacterial-based mock community. The mock communities were sequenced in duplicates and the number within brackets equals’ total number of unique identified or expected taxa in the samples.

Sequencing of the DNA-based mock community revealed that six out of eight bacterial species were correctly classified to the right genus. DNA from *E. coli* and *Salmonella enterica* were classified into the taxonomic family enterobacteriaceae and the taxonomic class gammaproteobacteria (Figure 3), which is the correct family and class, but the sequences were not classified to genus level. Sequencing of the bacterial-based mock community revealed that three out of five bacterial species were correctly classified to the right genus whereas *E. coli* and *Klebsiella pneumoniae* were classified into the taxonomic family enterobacteriaceae and the taxonomic class gammaproteobacteria (Figure 3), these sequences failed to be classified to genus level. Both in the bacterial-based and the DNA-based mock community we observed that there was a shift in the relative abundance among the included species compared to what was expected. The bacterial-based and DNA-based mock communities contained 5 and 8 species of bacteria respectively. Included bacterial species in the mock communities and their theoretical relative abundance are presented in Figure 3. After sequencing 5 and 7 bacterial taxa were identified in the bacterial-based mock community with high biomass (10^7^ and 10^5^ cells of each species per mL), and in the DNA-based mock community, 9 bacterial taxa were identified. More than 99% of the reads were associated with the input bacteria in the high biomass bacterial-based mock community and the DNA based mock-community. However, in the bacterial based mock community with 10^3^ cells of each bacterial species, the input bacteria only accounted for 55% (46% and 64% respectively in duplicates) of the total reads and in total 51 different taxa were identified in these samples (Figure 3). The pattern of more bacterial taxa in samples with lower microbial biomass was not observed in the milk samples where culture data was available (Additional file 1).

### Multivariate analyses of sequence data from milk samples

**Figure 4:**
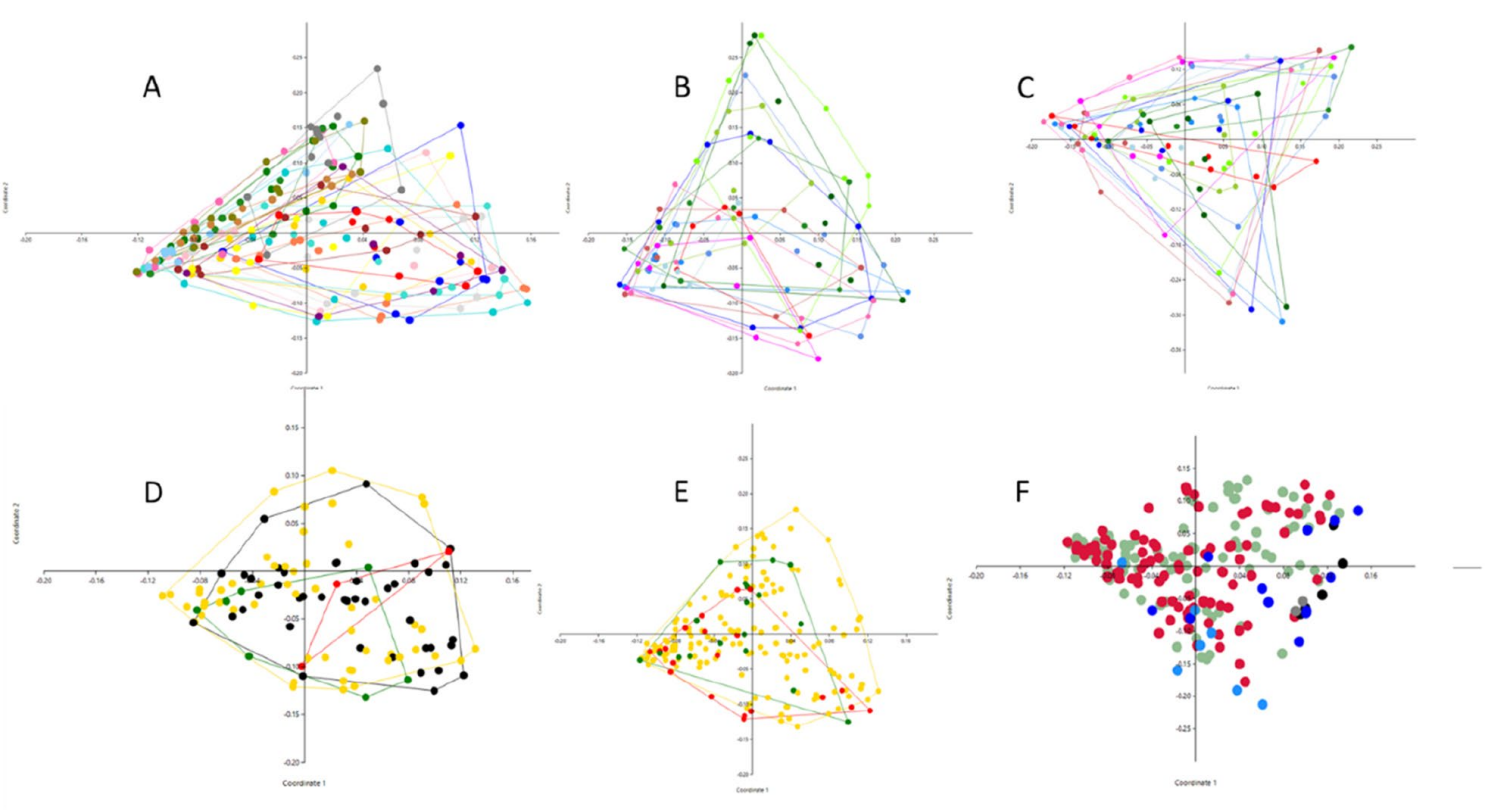
**Principal coordinate analysis plots based on Bray-Curtis distances on generated microbiota data.** In panel **A** coloring is based on individual cows, in panel **B** coloring is based on time in relation to infusion for control cows, in panel **C** time in relation to infusion for LPS-infused cows. In both panels **B** and **C**, blue color scale represents samples collected before infusion, green color scale represents samples collected 1–4 h post-infusion and the red color scale represents samples collected > 23 h post-infusion. Panel **D** is colored according to the amount of bacterial growth on plates (0 cfu (black colour), 1–10 cfu (yellow colour), 11–50 cfu (green colour) or > 50 cfu (red colour) per 100 µL of cultured milk after 48 h). In panel **E** grouping is based on SCC (< 150 000 (yellow colour), 150 000–1 000 000 (green colour) or > 1 000 000 (red colour) cells per mL) and in panel **F** all samples are included where green dots represents control cows, red dots; LPS infused cows, blue dots; negative controls (NTC and negPCR) and black dots mock communities.

**Figure 5:**
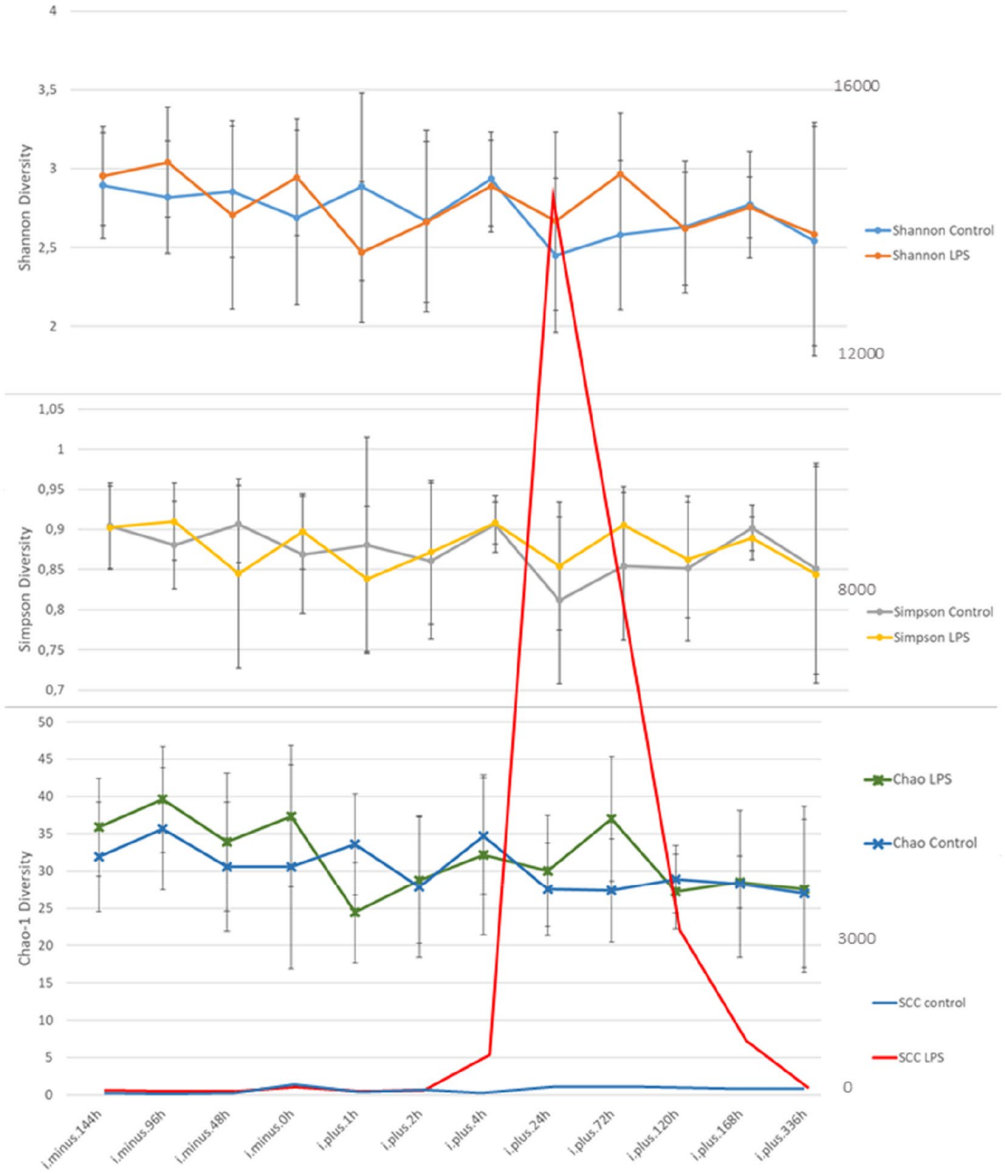
**Shannon, Simpson and Chao-1 diversity ± SD for control and LPS-infused quarters over time.** Mean SCC ×1000 cells per mL for control and LPS-infused quarters is superimposed with values on the secondary y-axis for visualisation.

A PCoA and ANOSIM based on Bray Curtis distances was applied to the data to search for clustering patterns among the milk samples, as well as similarity between milk samples and sequenced negative and positive controls (Figures 4A-F). Regardless of whether milk samples were grouped by SCC, bacterial growth in 100 µL of milk, or time in relation to infusion (analysed separately for control and LPS-infused cows), no clusters nor significant differences between groups could be identified (Figures 4B and E). The ANOSIM revealed that when grouped by individual cow (as seen in Figure 4A), some cows were significantly different in their microbiota profile compared to other cows (Additional file 2). Change in bacterial diversity over time was also analysed by comparing the Shannon, Simpson and Chao-1 diversity for control and LPS-infused cows (Figure 5) but no change related to inflammation could be identified.

### Data filtration

We used two different models in an attempt to identify contamination and filter out contaminating taxa from the data. In model 1; the R-package “decontam” was used and 42 different bacterial taxa were identified as contamination. In addition, 36 bacterial taxa were identified as contamination by their unexpected presence in the sequenced mock community and likewise excluded. A list of bacterial taxa identified as contaminants is presented in Additional file 3. The 78 bacterial taxa identified as contamination by data filtration model 1 contributed to 64.6% of all the reads in the data set, leaving 773 051 reads for further analysis. The number of reads available for further analysis after data filtration was not different between milk samples, NTCs or negPCR (3119 ± 1224, 2986 ± 2025 and 2293 ± 2428 reads per sample ± SD, respectively).

**Figure 6:**
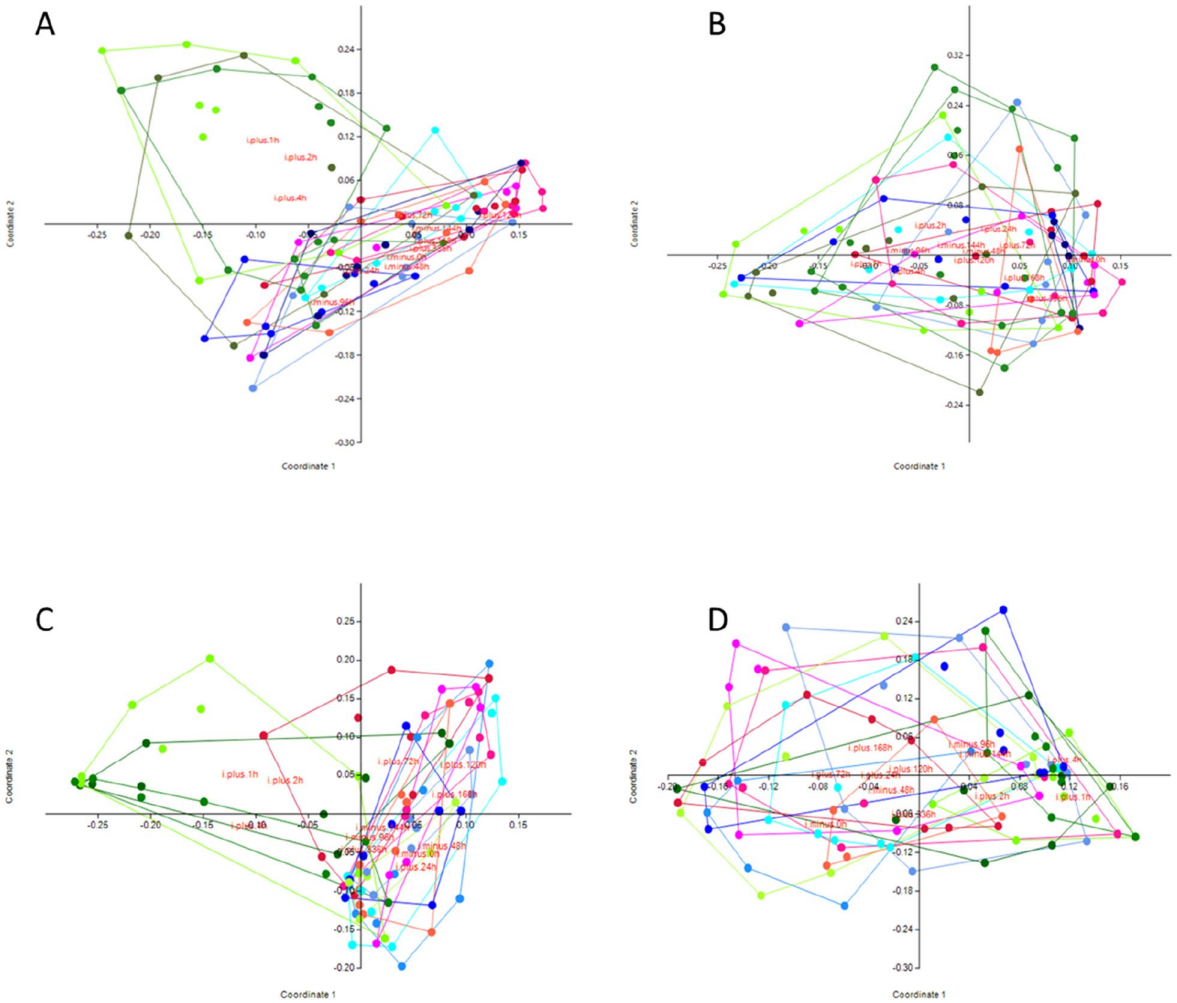
**Principal coordinate analysis plots with Bray-Curtis similarity index, grouped in relation to time of infusion after data filtration**. **A** and **B** after data filtration model 1, **C** and **D** after data filtration model 2. **A** and **C**; LPS infused cows, **B** and **D**; control cows. In all panels, samples collected before infusion are in blue colours, 1–4 h post-infusion in green colours and > 23 h post-infusion in red colours.

The genera *Enterococcus* and *Staphylococcus* were identified by the “decontam” R-package as contamination and were removed from the data set even though both genera were included in the mock communities. A PCoA based on Bray Curtis distances was applied to the filtered data to evaluate the effect of data filtration on clustering in relation to time of infusion for LPS infused and control cows (Figures 6A and B). Although a tendency of separate clustering for samples taken 1 h, 2 and 4 h after LPS infusion was noticed, a significant difference in the clustering pattern was only found for the sampling time 1 h post-infusion compared to 96 h before infusion, 48 h before infusion and 336 h post-infusion in ANOSIM using Bonferroni corrected *p*-values.

Using data filtration model 2, 99 different bacterial taxa had an abundance of > 1% in a negative control and were identified as contaminants and subsequently removed. Bacterial taxa identified as contamination with data filtration model 2 are presented in Additional file 3.

Data filtration according to model 2 had a substantial effect on the data, 88.0% of the available reads were discarded leaving 264 090 reads for further analysis. Eleven samples (3 milk samples, 8 negative controls) out of the 217 samples had no reads after filtration. After data filtration, according to model 2, 8 of the 11 species of bacteria in the mock communities were removed from the data set. Again, a PCoA based on Bray Curtis distances was applied to the filtered data to evaluate the effect of data filtration on clustering in relation to time of infusion for LPS infused and control cows (Figures 6 C and D). A similar tendency of separate clustering for samples taken 1 h, 2 and 4 h after infusion, as noticed in the PCoA after data filtration model 1, was observed. However, a significant difference in the clustering pattern was only found for the sampling time 4 h post-infusion compared to 0 h before infusion and 120 h post infusion in ANOSIM using Bonferroni corrected *p*-values.

**Figure 7:**
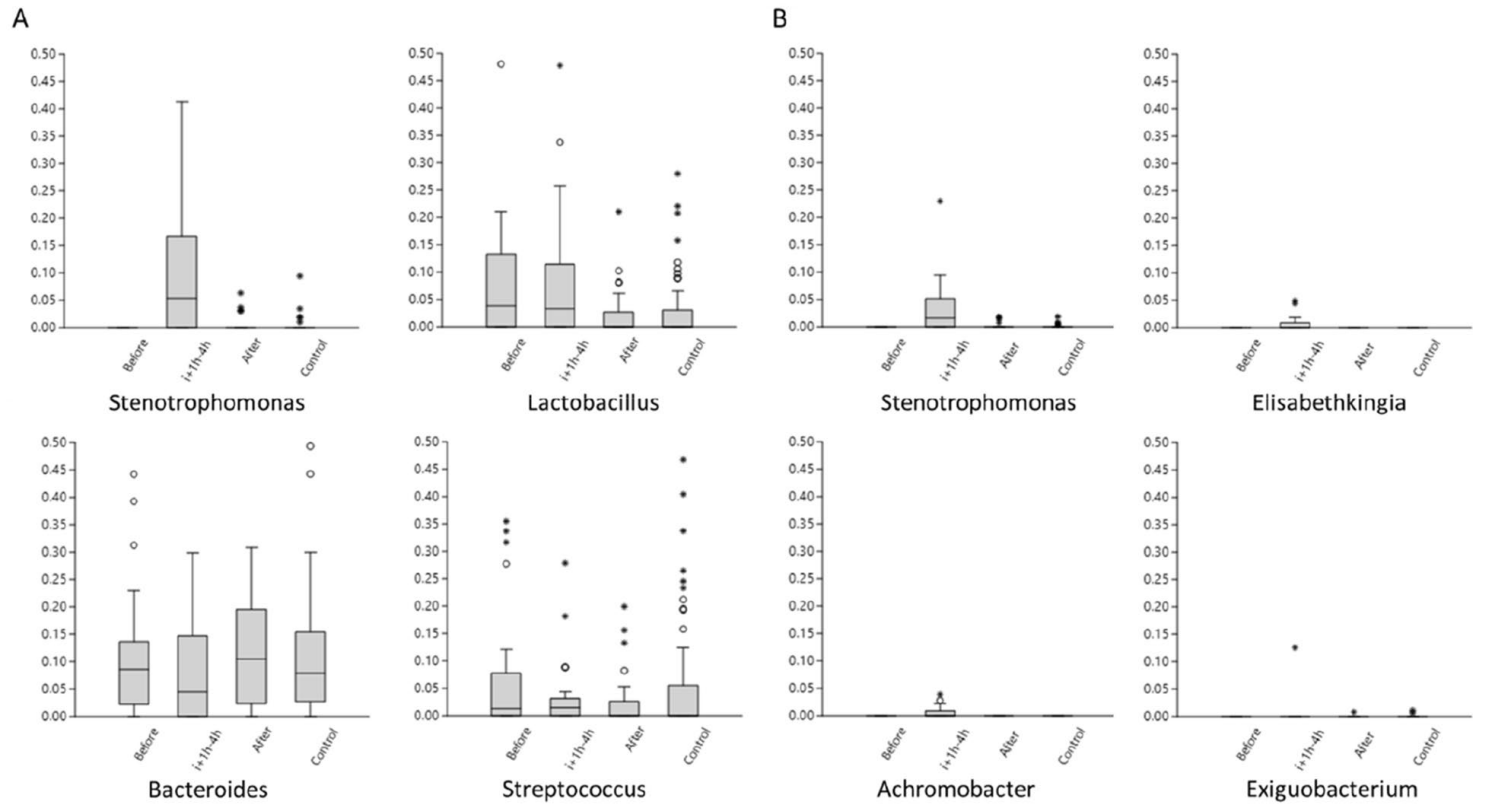
**Boxplot of the most abundant genera in samples taken 1 h, 2 and 4 h post-infusion after data filtration according to model 1 (panel A) and 2 (panel B) compared to relative abundance in samples taken before LPS infusion, > 23 h after LPS infusion and control cows.** Relative abundance on y-axis is truncated at 0.5 (50%). For boxes; the 25–75% quartiles are in the box, values > 1.5 box-height are shown as circles, values > 3 box-height are shown as stars.

In an attempt to explain what bacterial taxa that affected the variation after data filtration the relative abundance of the four most abundant genera in samples taken 1 h, 2 and 4 h after infusion was compared to the abundance in samples taken before infusion, samples taken more than 23 h after infusion and to the samples from control cows (Figure 7). *Stenotrophomonas* was the most abundant genus 1 h, 2 and 4 h post-infusion after data filtration with model 1 and 2. We noted that *Stenotrophomonas* was absent in the milk samples taken before LPS-infusion and had very low abundance in milk samples collected more than 23 h post LPS-infusion and in milk samples from control cows.

## Discussion

In this study it could not be shown that the strong inflammatory response to infusion of *E. coli* endotoxin is accompanied or followed by effects on the milk microbiota. From the obtained results it is clear that the data is strongly influenced by contamination. Contamination can have different origin and be introduced anywhere from sample collection to sequencing. In this study, samples were handled aseptically and efforts were made to avoid contamination (gloves were worn at sampling, sterile and DNA-free equipment were used in contact with the samples, DNA extraction and first PCR were performed on a chlorine cleaned, UV-irradiated LAF-bench) and controls were included to identify contamination introduced during DNA extraction and amplicon amplification. The included controls showed that a large proportion of bacterial taxa were introduced in the later parts of sample preparation.

Sequencing of negative controls from DNA extraction (NTC) and blank PCR reactions (negPCR) showed a large variation between samples and, surprisingly, many different bacterial taxa (Figure 2). Further, the bacterial composition found in negative controls showed a large overlap with the composition found in milk samples. A surprising discovery was that the number of reads per sample was higher in sequenced NTCs compared to milk samples. For the negPCR the average number of reads per sample and number of identified taxa per sample was lower than for milk samples and NTCs (Figure 2). Milk is known to contain PCR inhibitors such as calcium ions and plasmin [42]. It has also been shown that competing host DNA can significantly affect detection levels [43] in q-PCR analyses. Non-optimal conditions in the PCR reaction can thus be an explanation for these results. In addition, because of the low bacterial biomass in the milk samples, it was necessary to use many PCR-cycles in this study. For every PCR-cycle there is an increased risk of introduced PCR artefacts, which could affect the results. Thus, the number of PCR-cycles is a potential source for errors.

Sequencing of a bacterial-based mock community showed that the level of contamination increased with small bacterial biomass in the samples, however similar results was not observed in milk samples where culture data was available (Additional file 1). Even though 63% of the milk samples were culture positive (had at least 1 cfu after 48 h on 100 µL inoculated milk), the bacterial load in the cultured milk samples was very low, the absolute majority of the milk samples (117/120) had less than 500 cfu/mL and could thus be a likely explanation for the lack of comparable results.

Identification and filtration of contamination is a strategy used to acquire data that is unaffected by contamination [27]. In this study, identification of contamination and data filtration was used as a tool to assess our hypothesis that microbiota is affected by inflammation. Two different data filtration strategies were assessed, both models had a substantial effect on the data reducing number of available reads with 64% and 88%, respectively. The data filtration models were hampered by several limitations. Both models identified and filtered out bacterial taxa known to be present in the samples, i.e. taxa present in the mock communities. Moreover, the large variation of bacterial taxa identified among the blank controls made it more cumbersome to identify and filter out contamination. Nevertheless, both filtration models generated a more pronounced clustering of samples in relation to time of infusion and resulted in that sample points in relation to infusion became significantly different. When closer analysed *Stenotrophomonas* was the most abundant genus found in milk samples taken 1 h, 2 and 4 h after infusion after data filtration. The observation that *Stenotrophomonas* was absent in milk samples taken before infusion and barely detected in control cows indicate that DNA from *Stenotrophomonas* was infused together with the dissolved LPS.

The absence of correlation between SCC and milk microbiota is in line with previous reports from Derakhshani et al. [44] but in contrast to Ganda et al. [5, 22]. Also, the lack of response in Shannon diversity over the course of inflammation is in contrast to Ganda et al. [5, 22], (Figure 5). A reasonable conclusion for these results is that the bacterial source for inflammation but not LPS or the inflammation per se is the driving force for alteration of the microbial diversity.

In this study we hypothesised that (a) there is a microbiota in milk from healthy individuals that is affected by LPS-induced inflammation and that (b) data filtration might be needed in order to find differences related to inflammation in the milk microbiota. Our hypothesis that microbiota is affected by inflammation could not be confirmed. When samples were grouped based on SCC level or time in relation to infusion no differences between groups could be shown. For the second hypothesis, data filtration did indeed lead to visualization of differences between samples in relation to time of infusion but these differences were not in line with the development of clinical signs of inflammation or increased SCC. Consequently, we conclude that inflammation per se does not affect the microbiota in milk from healthy cows.

## Supplementary Information

**Additional file 1. Boxplot of number of bacterial taxa in milk samples and sequenced bacterial-based mock communities. **Samples are grouped by bacterial biomass, for milk samples this corresponds to number of cfu in 100µL of cultured milk and for mock communities from input number of bacterial cells. Number of bacterial taxa retrieved from rarefied data. No growth *n* = 41, 1-10 cfu *n* = 53, 11-50 cfu c = 7, >50 cfu *n* = 3, for all bacterial-based mock communities *n* = 2.

**Additional file 2. ANOSIM with BrayCurtis similarity index after grouping by individual cow. **PCoA of the same data is found in Figure 4A in the main text. Bonferroni corrected *p*-values above diagonal, R values below, significant differences marked with red.

**Additional file 3. List of bacterial taxa identified in negative controls and lists of bacterial taxa excluded in data filtration model 1 and 2.**

## Data Availability

Raw sequences have been submitted to the National Center for Biotechnology Information under Bioproject accession number PRJNA580390.
