# Supplementary figures and images for: Absence of changes in the milk microbiota during Escherichia coli endotoxin induced experimental bovine mastitis
Source: Vet Res. 2023 Jun 8;54:46. doi: 10.1186/s13567-023-01179-5 (PMC10251687; doi:10.1186/s13567-023-01179-5)

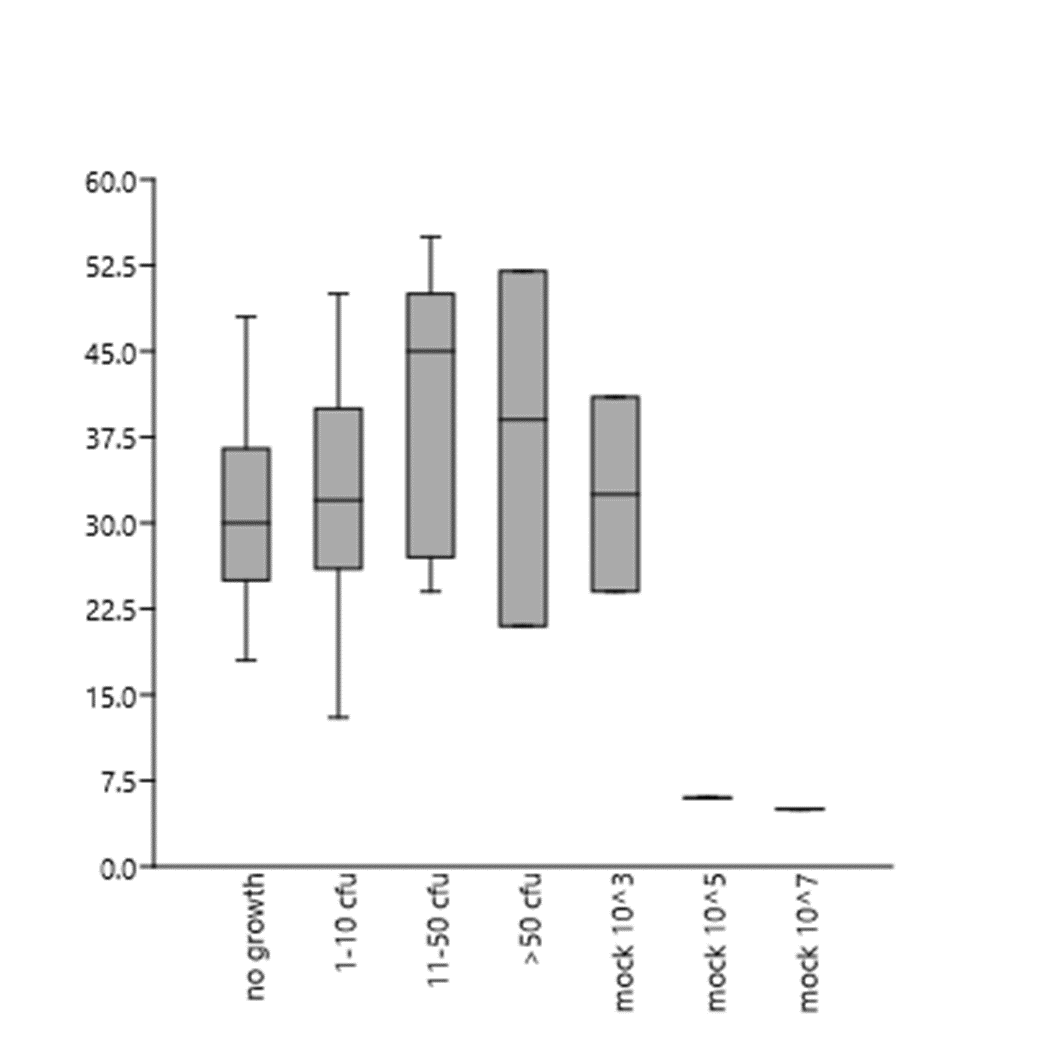

Supplement: Supplementary file 1 — Additional file 1. Boxplot of number of bacterial taxa in milk samples and sequenced bacterial-based mock communities. Samples are grouped by bacterial biomass, for milk samples this corresponds to number of cfu in 100µL of cultured milk and for mock communities from input number of bacterial cells. Number of bacterial taxa retrieved from rarefied data. No growth n = 41, 1-10 cfu n = 53, 11-50 cfu c = 7, >50 cfu n = 3, for all bacterial-based mock communities n = 2. [file 13567_2023_1179_MOESM1_ESM.docx]

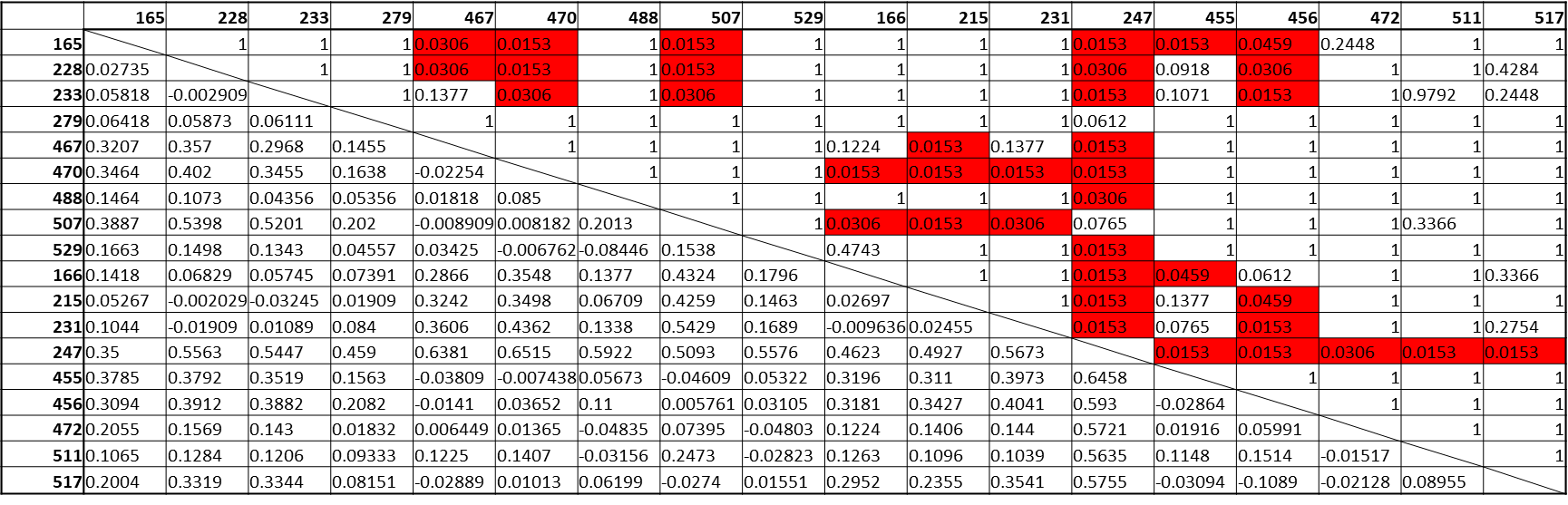

Supplement: Supplementary file 2 — Additional file 2. ANOSIM with BrayCurtis similarity index after grouping by individual cow. PCoA of the same data is found in Figure 4A in the main text. Bonferroni corrected p-values above diagonal, R values below, significant differences marked with red. [file 13567_2023_1179_MOESM2_ESM.docx]
